# Novel, Inexpensive Portable Respiratory Protection Unit (PRPU) for Healthcare Workers

**DOI:** 10.5811/cpcem.2020.4.47443

**Published:** 2020-04-15

**Authors:** Christopher S. Sampson, Adam Beckett

**Affiliations:** University of Missouri School of Medicine, Department of Emergency Medicine, Columbia, Missouri

**Keywords:** COVID-19, intubation, airway

## Abstract

**Introduction:**

Given concern for increased aerosolization during intubation of patients with severe acute respiratory syndrome coronavirus, we sought to create a portable, inexpensive, and easily constructed device to help protect healthcare workers.

**Methods:**

A respiratory protection unit can be constructed in approximately 30 minutes and for less than 50 United States dollars in materials, using polyvinylchloride pipe and automobile collision wrap.

**Conclusion:**

This device provides possible increased protection during video laryngoscopy and can easily be replicated.

## INTRODUCTION

Based on current evidence, the novel human coronavirus that is named severe acute respiratory syndrome coronavirus 2 (SARS-CoV-2) has been found during experimentation to remain viable when aerosolized for three hours.^1^ Current US Centers for Disease Control and Prevention guidelines recommend use of the highest level of personal protective equipment (PPE) when performing aerosolization-potential procedures such as intubation.^2^ Limited studies have shown a possible benefit of barrier protection when performing endotracheal intubation.^3^ We sought to create an inexpensive and easily reproducible model that could be used in resource-poor or resource rich-environments, or when negative pressure rooms are unavailable due to patient volumes.

## METHODS

The following materials were used to construct the portable respiratory protection unit (PRPU): polyvinyl chloride (PVC) ½ inch pipe; PVC joints; and 36-inch wide automobile plastic collision wrap (Table).

**Image 1:**
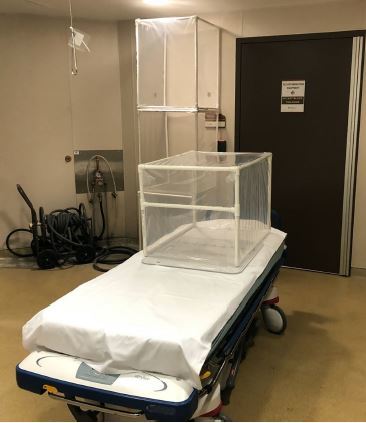
Portable respiratory protection unit, constructed of PVC pipe and auto collision wrap, situated on a stretcher in correct position.

PVC pipe can be purchased in one 10-foot piece that can be cut up into 13 pieces with a miter saw or hacksaw. Completed box dimensions are 24 inches tall and 27 inches in length. Three-way fittings are used on superior surface for the four joints and two 90-degree fittings are used on base corners. On the posterior (caudal) surface, two “T” fittings are used. Following frame construction, automobile collision wrap is used to cover each side. Collision wrap is often used in the automotive industry to cover broken car windows and is adhesive on one side. The adhesive side of the wrap faces into the box. Each box can be constructed in approximately 30 minutes by two people (Image 1). Total material cost is less than 50 US dollars.

**CPC-EM Capsule**

What do we already know about this clinical entity?

Given concern for healthcare worker exposure to aerosolization during intubation of the COVID+ patient, protective barriers have been suggested as a way to lessen risk.

What makes this presentation of disease reportable?

We present an inexpensive device that requires little construction.

What is the major learning point?

A portable respiratory protection unit can easily be replicated at low cost and used in the emergency department setting.

How might this improve emergency medicine practice?

Healthcare workers can be provided with some protection during airway management.

**Image 2:**
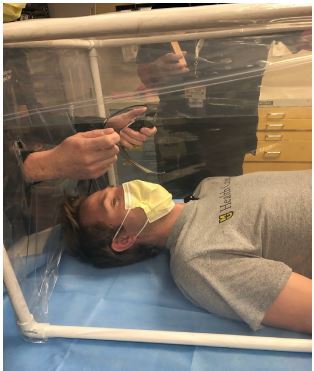
Simulated intubation through incisions made in cephalad wall of portable respiratory protection unit.

During intubation, the box is laid over the patient to cover his head and upper chest. Using a knife or any other sharp device, two vertical incisions can be made to place hands through (Image 2). Equipment can be passed through either the incisions or under the bottom of the plastic covering. Ideally the intubation being performed would be video laryngoscopy so that the healthcare worker would not be required to be close to the patient’s oropharynx. Following intubation, the box could be removed or ventilator tubing could be passed through incisions in the plastic. To limit ventilator circuit disconnection, tubing could be passed underneath the frame. Following use, the automobile collision wrap can be discarded and the frame can be cleaned according to CDC guidelines with wipes or sprayed down with appropriate cleaning agent. Once dry, a new length of automobile collision wrap can again be used to cover sides of the device in preparation for the next patient.

## DISCUSSION

The PRPU can be assembled rapidly and easily and materials should be readily available in most countries. If auto collision wrap is not readily available, a substitute plastic material could be used; however, depending on the opacity of the material it may further limit direct visualization of the patient. The PRPU use could be expanded beyond the emergency department setting. Other potential uses include protective covering for patients during emergency medical services transport or hospital transport.

## LIMITATIONS

Given the ever-changing situation and time-sensitive nature of disseminating this model, we do not have time to trial this model. By no means does this model completely contain aerosol viral particles; therefore, appropriate personal protective equipment should still be worn when intubating any known positive or suspected SARS-CoV-2 patient.

## CONCLUSION

The PRPU is an inexpensive and quick to construct a protective device for healthcare workers to use during intubation of high-risk patients such as those with SARS-CoV-2 The device is easy to reuse and has many additional applications.

## Figures and Tables

**Table:** Materials required to construct a portable respiratory protection unit.

Materials required
Miter saw or hacksaw 10 feet polyvinyl chloride ½-inch diameter pipe cut into these lengths: #4 28-inch length #3 22-inch length #2 20-inch length #2 7.5 inch (top bars) #2 11 inch (bottom bars) #2 ½-inch “T” fitting #4 90-degree fittings #4 3-way fitting 36-inch automobile plastic collision wrap (one roll)

## Appendix

Find the video at: https://vimeo.com/403079338.
